# The use of an invitational letter to increase the vaccine uptake of patients with coeliac disease

**DOI:** 10.1017/S1463423619000781

**Published:** 2019-10-30

**Authors:** Joseph Moneim, Hera Asad, Eman Butt, Jamil Shah Foridi, Yasmin Khan, Safwaan Patel, Jawad Qureshi, Ravi Thakar

**Affiliations:** School of Clinical Medicine, University of Cambridge, Cambridge, UK

**Keywords:** coeliac disease, invitation letter, vaccinations

## Abstract

**Aim:**

We sought to establish the impact on vaccine uptake of sending out a single appointment letter inviting patients to attend a vaccine clinic.

**Background:**

Coeliac disease is associated with splenic dysfunction and so patients with coeliac disease are at a higher risk of overwhelming infection. Additional vaccinations are recommended for these individuals to provide additional protection against infection.

**Methods:**

We retrospectively identified 54 patients with diagnosed coeliac disease, and all vaccines previously received by these patients. By comparing this to the Green Book [Department of Health (2013) Immunisation of individuals with underlying medical conditions: the green book, chapter 7, London: Department of Health. Retrieved 26 February 2019 from https://assets.publishing.service.gov.uk/government/uploads/system/uploads/attachment_data/file/566853/Green_Book_Chapter7.pdf], we determined the patients who were due vaccinations and the specific vaccines they were due. An invitation letter was then sent out to patients requiring further vaccinations and vaccine uptake for these patients was re-audited six months later.

**Findings:**

Our results show a mild increase in the total uptake of vaccines six months after the letter was sent out, from 38.6% to 49.2%.

## Background

Coeliac disease is a genetically determined chronic inflammatory disease of the small intestine where symptoms are induced by the environmental precipitant gluten. It affects approximately 1% of the population though a significant number remain undiagnosed (Lebwohl *et al.*, 2018). Coeliac disease is also associated with a number of extraintestinal symptoms, one of which being functional hyposplenism or splenic atrophy. The prevalence of hyposplenism in patients with coeliac disease is not well known with different investigators reporting an incidence of 21% to 60% (Halfdanarson *et al.*, 2007). It appears to be more common when coeliac disease coexists with other autoimmune conditions such as insulin-dependent diabetes mellitus and when there has been prolonged exposure to gluten. It is far less common in children (Corazza *et al.*, 1982; Di Sabatino *et al.*, 2013).

Functional hyposplenism is characterised by defective immune responses against infectious agents, particularly encapsulated organisms. This is because the spleen is crucial for the maturation and maintenance in the circulation of IgM memory B-lymphocytes. Encapsulated organisms are able to initially evade immunity mediated through T-lymphocytes and complement as their polysaccharide capsule hides the protein components of the cell membrane that would trigger their response. Thus, the elements of humoral immunity produced by the spleen are indispensable against these organisms. The spleen also produces opsonins which when attached to a microorganism will enhance phagocytosis by macrophages (Kirkineska *et al.*, 2014).

As well as an increased incidence of infections caused by encapsulated organisms, these infections can also be life-threatening and result in overwhelming post-splenectomy infection (OPSI) syndrome which is a septic syndrome characterised by a massive bacteraemia, no obvious primary source of infection, septic shock and disseminated intravascular coagulopathy. Studies have shown a relationship between coeliac disease and increased incidence of invasive pneumococcal disease (Röckert Tjernberg *et al.*, 2017); there have also been cases of fatal infections due to coeliac disease-associated hyposplenism (Simons *et al.*, 2018). Zingone *et al.* (2016) concluded in 2016 that unvaccinated patients with coeliac disease under the age of 65 had an increased risk of community-acquired pneumonia that was not found in vaccinated patients. They suggested that the low vaccination uptake in coeliac patients was a missed opportunity to protect patients from pneumonia. The commonest organisms associated with severe infection were pneumococcus (Streptococcus pneumoniae), Haemophilus influenzae type b and Neisseria meningitidis.

The Green Book detailing information on immunisations states that in the presence of a dysfunctional spleen, alongside the national schedule, these patients should be offered a vaccination against pneumococcal infection and an annual influenza vaccine due to the zrisk of secondary bacterial infection. It also recommends vaccination against haemophilus influenzae type b and against meningococcal types A, C, W, Y and B. There is currently limited literature on uptake of these vaccinations in the coeliac disease population. The aim of this audit was to initially establish the uptake of the above vaccinations within a single UK practice and then assess whether the use of a single invitation letter resulted in an increase in the vaccine uptake and thus number of patients up to date with vaccinations.

## Methods

Electronic patient records at a single merged UK general practice with a registered population of 25 859 were searched retrospectively using a single eligibility criterion of whether patients had a formal diagnosis of coeliac disease. Fifty-four (0.2%) patients were identified. Each patient’s immunisation history was then searched on 23 October 2017 to establish if they adhered to the following seven binary data points: pneumococcal vaccine (PPV23) in the last five years, influenza vaccine in the last year, haemophilus influenzae type b vaccine ever, meningococcal C vaccine ever, meningococcal groups A, C, W, Y vaccine ever (MenACWY), meningococcal group B vaccine (MenB) and a meningococcal group B booster dose one month after the first dose.

These specific immunisations are recommended in chapter 7 of the Green Book (Department of Health, 2013) which was last updated on 29 September 2016. They are intended for individuals with asplenia, splenic dysfunction or complement disorders. Since over 30% of adults with coeliac disease are thought to have defective splenic function (Corazza *et al.*, 1999), these patients are offered these vaccines routinely at the practice.

**Figure 1.**
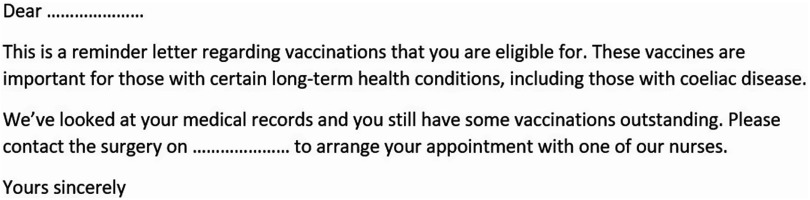
Picture of invitation letter sent to patients.

After establishing the number of patients that were due vaccinations and the specific types that they required, an invitation letter (Figure 1) was then sent to all patients who were not up to date with vaccines (54 patients) in November 2017. This coincides with the beginning of the UK flu season (December–March).

Data were then collected again on 27 March 2018 from patient records to ascertain the vaccination uptake in the cohort. This was then compared to the initial data to establish the effect of the invitation letter.

## Results

In October 2017, 54 patients were identified with coeliac disease at the practice; the majority (*n* = 32, 59%) were female patients and the age distribution of the cohort was between 18 and 86 years, with the mean age of 53 years. Invitation letters offering an appointment were sent to all 54 patients identified, as all required at least one vaccination, with the average patient due 4.30 vaccinations. All patients were due MenB (*n* = 54), 57.4% due influenza, 48.1% due MenACWY, 59.3% due PPV, 35.2% due HiB and 33.3% due MenC (Table 1). The total percentage vaccine uptake was 38.6%, as calculated by the formula: 1 – [total pending vaccines]/[total expected vaccines].

**Table 1.** Vaccination status of coeliac disease patients in October 2017 and April 2018

Vaccine recommended according to Green Book (Department of Health)	Patients up to date with vaccine (*n* = 54), *n* (%) in October 2017	Patients up to date with vaccine (*n* = 54), *n* (%) in April 2018
Meningitis B	2 (3.7)	19 (35.2)
Meningitis B 1/12 booster	0 (0.0)	11 (20.4)
HiB	35 (64.8)	37 (68.5)
Meningitis C	36 (66.7)	38 (70.4)
Pneumococcal ever	39 (72.2)	40 (74.1)
Pneumococcal up to date (five years)	22 (40.7)	19 (35.2)
Men ACWY (MENVEO)	28 (51.9)	31 (57.4)
Influenza ever	35 (64.8)	36 (66.7)
Influenza up to date (one year)	23 (42.6)	31 (57.4)

By April 2018, of the 54 coeliac disease patients, 25 (45%) attended an appointment for the vaccine clinic following recommendation by letter. From this intervention, the total percentage uptake of vaccines across all patients increased from 38.6% to 49.2%. The post-intervention percentage coverage per vaccination were as follows: MenB with booster 20.4%, Influenza 57.4%, MenACWY 57.4%, PPV 35.2%, HiB 68.5%, MenC 70.4% (Table 1). Furthermore, by April 2018, 14.8% (*n* = 8) patients were fully up to date with all their vaccinations related to management of their coeliac disease.

## Discussion

The aim of this audit was to examine whether a single letter inviting patients with coeliac disease to book an appointment for vaccines was effective in increasing vaccine uptake. Our results show a 10.6% increase in total vaccine uptake five months after the letter was sent out. This indicates that patients did to a certain extent respond to the letter and that in this cohort it was an effective prompt to increase vaccine uptake. However, it was not completely successful as after the letters had been sent only 46% attended the surgery and thus a number of people had ignored the prompt. Additionally, only eight patients were fully up to date at the end of the study. This may be due to a large variation in public perception of vaccines and their necessity. In Europe, 82% of people declared a positive opinion on vaccinations; however, of this number 45% indicated the more reserved ‘somewhat positive’ stance (Schmitt *et al.*, 2007). Coeliac patients who diligently avoid gluten remain well and while well, they may feel it unnecessary to receive extra vaccinations.

Another interesting finding was the differing uptakes of different vaccinations. The most marked rise in vaccine uptake was the meningitis B and the meningitis B one-month (1/12) booster. The meningococcal B (MenB) vaccination was only introduced into the UK national infant immunisation programme from 1 September 2015 where infants were offered a reduced two-dose primary immunisation at 2 and 4 months followed by a booster at 12 months (Ladhani *et al.*, 2015). Thus, it is likely that a large proportion of our patients with coeliac disease whose age ranged from 18 to 86 years will have never come across this vaccine before. Another large increase in uptake was an up-to-date Influenza vaccination, with the proportion of patients who were up to date increasing from 42.6% to 57.4%. This increase in uptake could be partially attributed to media coverage and recent flu pandemic scares. All the other vaccinations experienced modest increases in uptake except for an up-to-date pneumococcal vaccine which actually experienced a decrease in the number of people up to date over the six months (40.7% to 35.2%). At the practice, there was a previous attempt to increase vaccine uptake nearly exactly five years ago, and so a disproportionate number of patients were due the five-year booster vaccine within our audit cycle. This could explain the measured decrease in patients being up to date with the pneumococcal vaccine.

The letter did seem to be at least partially effective as described above. This was, however, a small sample in a single practice and it is difficult to assess whether the audit reflects the wider population. A potential confounding factor is that this study was conducted during the UK flu season, which may itself alter the awareness of patients about flu vaccination and may independently increase vaccination rates, as well as affecting the responses to letter invitation. It may be useful to repeat this study away from the flu season to better single out the effects of the intervention.

There is limited data on the impact of letters inviting patients for vaccinations. However, letters have been shown to be effective in inviting patients to NHS health checks for cardiovascular risk assessment (Sallis *et al.*, 2016) and inviting mental health patients for primary care physical health checks (Hardy and Gray, 2012). Public Health England has produced an invitation letter template for the flu vaccine for at-risk patients and NICE guidelines encourage awareness of eligibility possibly through the use of letters. We live in an increasingly modern society where the primary means of contacting people is changing from letters to electronic methods of communication. The use of texts or emails to contact patients requiring vaccinations is another avenue which could be investigated. SystmOne has a feature to automatically text people which could be utilised, and this would be cost-effective and convenient for patients who could call to book an appointment on their phones.

## Conclusion

Vaccines are an essential public health strategy to protect at-risk patients such as those with coeliac disease from serious consequences of infection by encapsulated organisms. We have shown that a simple intervention such as a single reminder letter can increase vaccine uptake in these patients.
